# Mid-term results of TAVI in high-risk patients: data from a single center study

**DOI:** 10.1186/1749-8090-8-S1-O315

**Published:** 2013-09-11

**Authors:** R Akchurin, T Imaev, P Lepilin, A Kolegaev, A Komlev, I Pokidkin

**Affiliations:** 1 Cardiovascular Surgery, Russian Cardiology Research Center Moscow, Russian Federation

## Background

Transcatheter aortic valve implantation (TAVI) has become a method of choice in repair of valvular aortic stenosis, especially in group of patients with high surgical risk.

## Aim

The objective was to evaluate the immediate and mid-term results of applying TAVI in aortic valve surgery.

## Methods

80 patients with a median age of 78 ± 5.2 years were included. All patients had severe aortic stenosis. According to the echo an average gradient of systolic pressure on the aortic valve was >20% by EuroSCORE and > 10% by STS. We have implanted Edwards Sapiens/Sapiens XT or Medtronic CoreValve bioprostheses. In 58 cases implantation was performed through transfemoral access (including 30 Edwards Sapien cases) and in 17 cases- transapical access was used due to vascular abnormalities. In the rest 5 patients we used direct transaortic (4 cases) and in 1 case - transsubclavian approach.

## Results

Intraoperative mortality was 1.25%: 1 patient (women) died with symptoms of acute heart failure. Total 30 days mortality rate was 5%: 2 patients died within 7 days after TAVI - IM and other 2 patients developed cardiogenic shock. Incidence of non-fatal stroke was 2.5%. In 2 patients the procedure of hemodialysis needed to be performed because of acute contrast-induced renal injury. Other patients had no significant complications. The AMPG after Edwards Sapien and Medtronic CoreValve implantations were 10,9±3,5 and 14,3±5,4 mm Hg respectively. The incidence of paravalvular leak of 2 degree had a tendency to be higher in CoreValve than in Edwards Sapien subgroup (15% vs. 5%). Nevertheless, by the end of 1-year follow-up only 2 patients had residual aortic regurgitation >2 degree (CoreValve). In 5 patients after CoreValve implantation (25%) - need of permanent pacemaker. The overall mortality rate by the end of 1-year was 12% with no procedure-related deaths.

## Conclusion

TAVI can be considered as a real alternative to traditional aortic valve replacement in high-risk patients.

